# Prediction of cognitive response to surgery in elderly patients with primary hyperparathyroidism

**DOI:** 10.1093/bjsopen/zraa029

**Published:** 2020-12-22

**Authors:** A Koman, R Bränström, Y Pernow, R Bränström, I -L Nilsson

**Affiliations:** Department of Endocrine Tumours and Sarcoma, Karolinska University Hospital, Stockholm, Sweden; Department of Molecular Medicine and Surgery, Karolinska Institutet, Stockholm, Sweden; Department of Endocrine Tumours and Sarcoma, Karolinska University Hospital, Stockholm, Sweden; Department of Molecular Medicine and Surgery, Karolinska Institutet, Stockholm, Sweden; Department of Molecular Medicine and Surgery, Karolinska Institutet, Stockholm, Sweden; Department of Clinical Neuroscience, Karolinska Institutet, Stockholm, Sweden; Department of Endocrine Tumours and Sarcoma, Karolinska University Hospital, Stockholm, Sweden; Department of Molecular Medicine and Surgery, Karolinska Institutet, Stockholm, Sweden

## Abstract

**Background:**

Primary hyperparathyroidism (pHPT) can be associated with potentially reversible cognitive impairment, which is occasionally mistaken for natural ageing and dementia. The aim was to evaluate short-term medical normalization of hypercalcaemia in surgical decision-making for elderly patients with mild cognitive deficiency.

**Methods:**

Patients with pHPT were included in a prospective observational study. A test panel including the Montreal Cognitive Assessment (MoCA) and validated tools for estimation of psychological status (Hospital Anxiety and Depression Scale, HADS), and muscle strength (timed-stands test, TST) was applied at baseline, after 4 weeks of calcimimetic treatment, and after parathyroidectomy. Mild cognitive impairment was defined by a MoCA score below 26. A longitudinal increase in MoCA score of at least 2 points 6 months after surgery was considered clinically meaningful.

**Results:**

Of 110 patients who underwent testing, 35 aged 50 years or more were identified to have mild cognitive dysfunction, including 19 who were aged at least 70 years (median MoCA score 23, i.q.r. 21–24). Calcimimetic treatment resulted in normalization of calcium levels, and improvements in MoCA and HADS scores, and TST time. Normal MoCA scores (at least 26) were reached in 17 patients by 6 months after surgery, of whom 10 were aged 70 years or older. Long-term increase in MoCA score correlated with the decrease in ionized calcium concentration (*r* = –0.536, *P* = 0.022). Baseline calcium concentration and improvement in MoCA with calcimimetic treatment were identified as independent predictors of favourable outcome after parathyroidectomy.

**Conclusion:**

Medical normalization of hypercalcaemia can aid in predicting outcome after parathyroidectomy.

## Teaser

Cognitive impairment related to primary hyperparathyroidism in the elderly is sometimes mistaken for natural ageing or dementia. The aim of this study was to evaluate the ability of medical normalization of hypercalcaemia to predict individual outcome after parathyroidectomy. Half of the 35 patients with mild cognitive deficiency (Montreal Cognitive Assessment (MoCA) score below 26) had normal MoCA scores (at least 26) by 6 months after operation. The improvement in MoCA score during receipt of study medication was identified as independent predictor of favourable outcome after parathyroidectomy.

Medical treatment predicts cognitive improvement after surgery

## Introduction

Primary hyperparathyroidism (pHPT) is characterized by a relative excess of parathyroid hormone with hypercalcaemia. The cause is most commonly a benign parathyroid adenoma, and the only curative treatment is parathyroidectomy. Conservative treatment can be adopted in asymptomatic disease, or calcimimetic treatment used to reduce symptoms of hypercalcaemia when surgery is contraindicated^1^. The incidence of pHPT increases with age and the disorder is three times more common in women. The prevalence is estimated to be around 1 per cent in the general Swedish population, but as high as 3–5 per cent among women aged over 50 years^2^.

Many patients with pHPT exhibit biochemically mild disease without classical features such as renal stones or osteoporosis. Many are classified as asymptomatic, although, particularly in the elderly, cognitive impairment, muscular weakness, diffuse neuropsychiatric symptoms, and pain are often mistaken for other age-related conditions. According to current guidelines, the majority of elderly patients with non-specific symptoms only do not fulfil the criteria for parathyroidectomy and are consequently at risk of not being offered curative treatment^3^^,^^4^. Persisting disease may lead to worsening symptoms and impaired quality of life (QoL) that potentially pose an increased risk of development of secondary chronic morbidity and disabilities. In the elderly patient with signs of cognitive deficiencies, the impact of parathyroidectomy on cognitive impairment and the potential of reversal is largely unknown.

Previous studies have demonstrated that surgical cure rates and clinical outcomes, including morbidity and mortality, are similar in the elderly to those among younger patients. Besides increased fracture-free survival after parathyroidectomy, the majority of elderly patients with pHPT experience general symptomatic relief^5^^,^^6^. Although overall long-term recovery from non-specific symptoms after parathyroidectomy has been established in both normocalcaemic and hypercalcaemic patients with pHPT^7^, with increasing age, elderly patients are less frequently referred for parathyroidectomy^5^. Predicting the outcome of non-specific symptoms and cognitive performance after parathyroidectomy remains a crucial question.

Short-term medical normalization of hypercalcaemia has been recognized as a feasible diagnostic method that might be used to predict the overall effects on QoL, cognition, and muscle strength after parathyroidectomy to aid in the decision to offer surgical treatment. A study^8^ confirmed that symptom relief achieved by medical normalization of hypercalcaemia correlated well with improvements after surgery. The calcium-lowering effect is almost immediate and reversed rapidly when the drug is discontinued. In a study^9^ of 22 patients with pHPT who received 30–50 mg cinacalcet twice daily, normal calcium levels were achieved after only the second dose on day 1, whereas another study^10^ with the same agent reported normalization of hypercalcaemia among 14 of 17 patients with intractable pHPT. Although adverse effects were common, a significant improvement in QoL and cognitive functioning was observed^10^. There is, however, no convincing evidence for long-term effects of calcimimetic treatment on non-specific symptoms, or its effects on risk reduction for cardiovascular complications or prevention of osteoporosis. Based on its described properties, cinacalcet was considered to be a highly suitable drug for trial purposes to see whether short-term outcomes might predict long-term changes in cognitive function. This study therefore sought to evaluate short-term calcimimetic treatment as a diagnostic tool to predict outcome after parathyroidectomy in elderly patients with pHPT and mild cognitive impairment.

## Methods

Factors influencing recovery after normalization of hypercalcaemia were analysed in a prospective observational study of patients with pHPT, who were treated with a short-term calcimimetic as a diagnostic tool to predict outcome after parathyroidectomy (ClinicalTrials.gov, NCT00982722). Some of the results have been presented previously, including a detailed description of the study procedure^8^. Each patient gave written consent to participate in the study, which was approved by the Swedish Medical Products Agency and the regional ethics committee. This part of the study focused on patients in the cohort aged 50 years or more with mild cognitive impairment as defined below.

Patients received calcimimetic treatment for 4 weeks, followed by parathyroidectomy. Treatment was initiated with 30 mg cinacalcet hydrochloride daily (Mimpara^**^®^**^; Amgen, Thousand Oaks, California, USA)^11^. If hypercalcaemia persisted after 2 weeks of daily 30 mg cinacalcet, the dose was increased to 60 mg. Calcium levels and side-effects were monitored and recorded weekly during this treatment. BP and levels of ionized calcium were recorded at all other assessments as detailed below.

A panel of validated tests to estimate cognitive function, mental status, and muscle strength was applied four times: at baseline, after 4 weeks of calcimimetic medication, and at 6 weeks and 6 months after parathyroidectomy. No specific length of time was mandated for the interval between cessation of calcimimetic treatment and the date of surgery. Test results were analysed longitudinally in order to compare the effect of normalization of hypercalcaemia with the result after parathyroidectomy. Positive (PPVs) and negative (NPVs) predictive values were calculated for each test.

The Montreal Cognitive Assessment (MoCA)^12^^,^^13^, a validated screening instrument for early screening of cognitive impairment, was used. Normative scores for the MoCA, based on a large Swedish population of individuals aged 65–85 years, have been published^14^. Visuospatial and abstraction capacity, memory, executive capability, attention, language, and orientation in time and space are evaluated. The maximum score is 30 points. Age and education have an impact on the results. For individuals with 12 years or less of education, 1 point is added to the total score. A score of below 26 points is established as the cut-off value for indicating mild cognitive impairment. Alzheimer’s disease should be considered for a score of less than 22 points.

The Hospital Anxiety and Depression Scale (HADS) questionnaire includes six items for the detection of depression (HADS D) and six for anxiety (HADS A)^15^. Each item is scored on a scale of 0–4, lower scores being favourable.

The timed-stands test (TST) is used to estimate proximal muscle strength by measuring the time required to complete 10 full stands from a sitting position as quickly as possible. The time is rounded off to the nearest tenth of a second. A reduction in time spent performing the test indicates an improvement in proximal muscle strength^16^.

Blood samples were collected with the patient in a fasting state before each visit. All biochemical analyses were performed using routine methods by the Karolinska University Laboratory in Stockholm. Blood analysed for ionized calcium was delivered within 1 h, and centrifuged within 2 h of collecting the sample.

Glomerular filtration rate was calculated from preoperative baseline blood samples using the Modification of Diet in Renal Disease formula: 175×(serum creatinine/88.4)^−1.154^×(age)^−0.203^×(0.742 if female).

### Statistical analysis

Based on the distribution, non-parametric methods were used and data presented as median (i.q.r.). All tests were two-tailed, and *P *<* *0.050 was considered statistically significant. Each patient served as their individual control. Wilcoxon signed-rank test was used for intraindividual comparisons. The Spearman rank-order test was used to analyse bivariable correlations. The improvements in each test were transformed into dichotomous variables. For the subgroup with mild cognitive impairment, cross-tabulation was used for calculating sensitivity, specificity, PPV, and NPV for each test. The area under the curve was estimated by receiver operating characteristic (ROC) curve analysis. A binary logistic regression model, including the whole study cohort, was used for analysis of predictors of long-term increase in MoCA score by at least 2 points, as defined by the test developer to be clinically significant^17^. Statistical analyses were done using SPSS^®^ version 26 (IBM, Armonk, New York, USA)

## Results

**Fig. 1:**
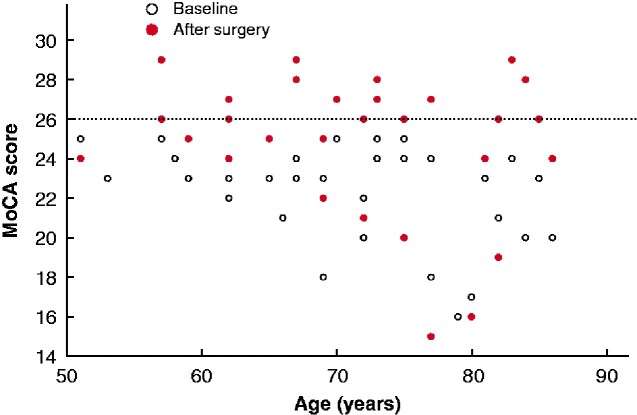
Montreal Cognitive Assessment score before and 6 months after parathyroidectomy in relation to age Scores on or above dotted line are considered normal. MoCA, Montreal Cognitive Assessment.

Clinical characteristics of the study cohort and subgroups are shown in *Table 1*. Of 110 patients enrolled, mild cognitive dysfunction, defined by a MoCA score below 26 (median 23, i.q.r. 21–24), was identified in 35 patients aged 50 years or more (19 patients aged at least 70 years). Overall, 17 of 35 patients (10 aged at least 70 years) had normal MoCA scores of 26 or higher 6 months after surgery (*Fig. 1*). A binary logistic regression model identified an increase in MoCA score during calcimimetic treatment and the baseline ionized calcium level, classified into tertiles, as independent predictors of long-term improvement in MoCA score (*Table S1*).

**Table 1:** Clinical characteristics

	All ages and MoCA scores^†^ (*n *=* *110)	MoCA score < 26, age ≥ 50 years (*n *=* *35)	MoCA score < 26, age ≥ 70 years (*n *=* *19)	Normal reference range
**Age (years**)^*^	62 (53–72)	71 (62–79)	77 (72–82)	
**Sex ratio (F : M)**	91 : 19	31 : 4	17 : 2	
**BMI (kg/m^2^**)^*^	26 (23–29)	27 (23–30)	26 (21–30)	
**Biochemical measurements** ^*^				
Plasma calcium (mmol/l)	2.60 (2.51–2.68)	2.62 (2.49–2.71)	2.67 (2.56–2.73)	2.15–2.50
Serum ionized calcium (mmol/l)	1.41 (1.38–1.46)	1.42 (1.39–1.48)	1.42 (1.38–1.48)	1.15–1.33
Plasma albumin (g/l)	38 (36–39)	38 (35–39)	36 (34–39)	36–48 (< 41 years) 36–45 (41–70 years) 34–45 (> 70 years)
Plasma parathytroid hormone (pmol/l)	10.0 (7.9–13.0)	11.0 (8.8–14.2)	11.0 (8.1–14.0)	1.6–6.0
Plasma phosphate (mmol/l)	0.86 (0.78–0.95)	0.85 (0.78–0.95)	0.83 (0.78–0.93)	0.8–1.5
Plasma creatinine (μmol/l)	68 (62–78)	69 (61–81)	69 (60–81)	< 90 (F) < 100 (M)
Serum 25-hydroxy vitamin D (nmol/l)	66 (52–81)	65 (49–73)	66 (50–76)	50–250
Thyroid-stimulating hormone (mEq/l)	1.8 (1.2–2.6)	1.8 (1.1–2.6)	1.6 (1.1–2.4)	0.3–4.2
Fasting blood glucose (mmol/l)	5.6 (5.3–5.9)	5.6 (5.2–6.1)	5.5 (5.0–6.4)	4.0–6.0
**Systolic BP (mmHg)** ^*^	142 (130–154)	148 (139–175)	151 (134–180)	
**Diastolic BP (mmHg)** ^*^	88 (82–96)	92 (84–100)	90 (80–100)	
**Increased dose of calcimimetic**	39	15	9	
**Adenoma weight (mg)** ^*^	356 (260–633)	468 (300–1000)	370 (322–925)	

* Values are median (i.q.r.).

† Montreal Cognitive Assessment (MoCA) score 26 (i.q.r. 24–28).

All but three of the 35 patients with mild cognitive impairment completed the study. Two patients stopped the medication because of gastrointestinal side-effects and one discontinued medication after an upper respiratory tract infection. Parathyroidectomy was performed at a median of 6 (i.q.r. 4–12) weeks after the last dose of study medication.

A total of 15 patients (9 aged at least 70 years) required higher-dose calcimimetic treatment (60 mg/day) to achieve normal calcium levels. No serious adverse event related to the study medication was observed. Calcimimetic treatment resulted in normalization of calcium levels and improvements in MoCA score (change compared with baseline in patients aged 50 years or more: median 2, i.q.r. 0–3; *P *<* *0.001), HADS score (–2, 0 to –3; *P *=* *0.020) and TST (–3 (0 to -9) s; *P *=* *0.004) (*Table 2*). The baseline MoCA score correlated inversely with age (*r* = –0.430, *P* = 0.010) but not with the ionized calcium level (*r* = 0.079, *P *=* *0.651).

**Table 2:** Test results in patients aged at least 50 years with a Montreal Cognitive Assessment score below 26 at baseline

	Baseline	△I–II	***P* (I *versus* II)** ^*^	△I–IV	***P* (△I–II *versus* △I–IV)** ^*^
**Age ≥ 70 years (*n* = 19)**					
MoCA score	22 (20–24)	1 (0–3)	0.004	2 (0–4)	0.358
Timed-stands test (s)	32 (26–45)	–3 (1 to –9)	0.061	–4 (–2 to –15)	0.006
HADS anxiety score	4 (2–10)	–1 (0 to –3)	0.505	–1 (0 to –3)	0.189
HADS depression score	4 (1–8)	–1 (0 to –3)	0.053	–1 (0 to –3)	0.189
HADS total score	8 (5–15)	–2 (0 to –3)	0.095	–3 (0 to –6)	0.073
**Age ≥ 50 years (*n* = 35)**					
MoCA score	23 (21–24)	2 (0–3)	< 0.001	2 (1–4)	0.091
Timed-stands test (s)	33 (28–40)	–3 (0 to –9)	0.004	–5 (–2 to –10)	0.033
HADS anxiety score	6 (4–10)	–1 (0 to –2)	0.140	–2 (0 to –3)	0.040
HADS depression score	4 (1–6)	–1 (0 to –2)	0.019	–3 (0 to –6)	0.040
HADS total score	8 (6–16)	–2 (0 to –3)	0.020	–3 (0 to –6)	0.005

Values are median (i.q.r.). Intraindividual changes are shown: ΔI–II, change between baseline (measurement I) and after 4 weeks of calcimimetic treatment (measurement II); ΔI–IV, change between baseline and 6 months after parathyroidectomy. MoCA, Montreal Cognitive Assessment; HADS, Hospital Anxiety and Depression Scale.

* Wilcoxon signed-rank test.

The predictive values of tests in the panel are presented in *Table 3*. HADS had the highest accuracy with PPV and NPV around 90 per cent. MoCA and TST had PPV values around 80 per cent, but an NPV of no more than 33 per cent.

**Table 3:** Prediction of improvement at follow-up 6 months after parathyroidectomy in patients aged at least 50 years and with a Montreal Cognitive Assessment score below 26 at baseline

	PPV (%)	NPV (%)	Sensitivity (%)	Specificity (%)	**AUC for**Δ**I–II**
**Age ≥ 70 years (*n* = 19)**					
MoCA score	82	33	69	50	0.596 (0.263, 0.929)
Timed-stands test	89	25	73	50	0.569 (0.209, 0.930)
HADS anxiety score	94	56	68	90	0.669 (0.394, 0.945)
HADS depression score	80	88	89	78	0.833 (0.629, 1.000)
HADS total score	90	100	100	89	0.944 (0.891, 1.000)
**Age ≥ 50 years (*n* = 35)**					
MoCA score	86	22	72	40	0.560 (0.273, 0.847)
Timed-stands test	94	33	74	75	0.745 (0.474, 1.000)
HADS anxiety score	94	56	68	90	0.791 (0.626, 0.956)
HADS depression score	75	83	88	67	0.775 (0.603, 0.946)
HADS total score	80	92	94	75	0.846 (0.700, 0.991)

Values in parentheses are 95 per cent confidence intervals. The predictive values of the different components of the test protocol were evaluated dichotomously based on improvement or not. Sensitivity, specificity, and positive (PPV) and (NPV) predictive values were calculated by cross-tabulation. AUC, area under the receiver operating characteristic (ROC) curve; ΔI–II, change between baseline and after 4 weeks of calcimimetic treatment; MoCA, Montreal Cognitive Assessment; HADS, Hospital Anxiety and Depression Scale.

**Fig. 2:**
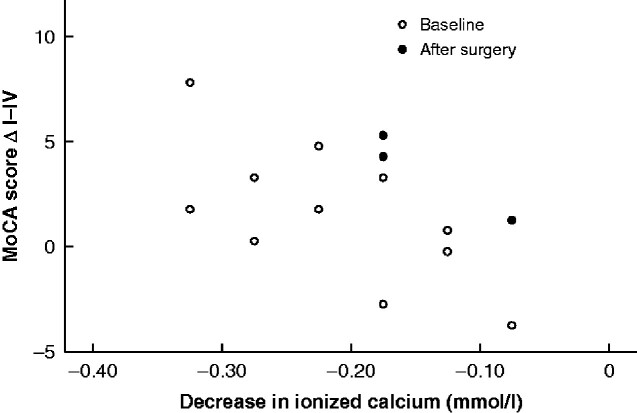
Change in Montreal Cognitive Assessment score 6 months after parathyroidectomy in relation to decrease in ionized calcium level MoCA, Montreal Cognitive Assessment; ΔI–IV, change between baseline and 6 months after parathyroidectomy.

For patients aged at least 70 years, long-term improvement in MoCA score after parathyroidectomy correlated with the decrease in ionized calcium level (*r* = –0.536, *P* = 0.022) (*Fig. 2*).

**Fig. 3:**
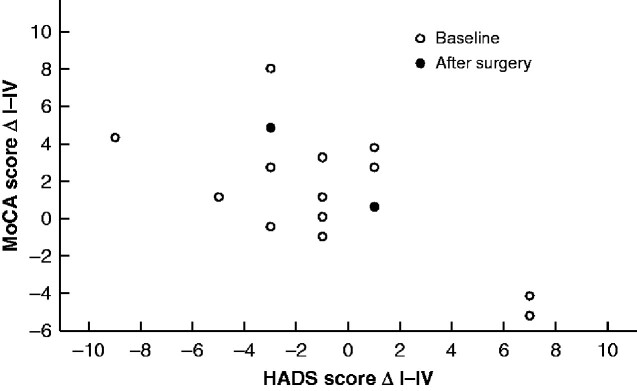
Change in Montreal Cognitive Assessment score 6 months after parathyroidectomy in relation to change in Hospital Anxiety and Depression Scale score during calcimimetic treatment MoCA, Montreal Cognitive Assessment; HADS, Hospital Anxiety and Depression Scale; ΔI–II, change between baseline and after 4 weeks of calcimimetic treatment; ΔI–IV, change between baseline and 6 months after parathyroidectomy.

The decrease in HADS during calcimimetic treatment correlated with an increase in MoCA score 6 months after parathyroidectomy (*r* = –0.488, *P* = 0.047) (*Fig. 3*).

## Discussion

Preservation of cognition, psychological functioning, and muscular strength are important in maintaining QoL and health in the elderly. Exclusion of patients from surgery who exhibit these non-specific manifestations of pHPT is of concern.

It is well recognized that calcimimetic treatment is effective in normalizing calcium levels in pHPT^18^, but adverse effects are frequent and the effect on the calcium concentration lasts only during continuous medication. Short-term normalization of hypercalcaemia has been suggested as a feasible diagnostic tool to predict outcomes after parathyroidectomy. The present study has confirmed that short-term improvements in cognitive function achieved in this way can be maintained for at least 6 months after surgery.

All assessments included in the test panel were performed easily and generated high predictive values regarding long-term benefits of parathyroidectomy. The MoCA screening test, which has been well validated for the detection of cognitive deficiency, and considered appropriate as a repeatable brief measure of longitudinal cognitive change^13^^,^^19^, indicated that half of the patients with a baseline score of less than 26 had normal scores 6 months after operation, irrespective of age. These results demonstrate the potential benefit on cognitive function, and underline the importance of establishing a correct indication for parathyroidectomy.

The short-term improvement in depression and anxiety after medical normalization of hypercalcaemia was also found to be associated with long-term effects on cognitive function 6 months after parathyroidectomy. This adds further support for consideration of non-specific symptoms in future guidelines for parathyroid surgery.

Ionized calcium is considered to be the most appropriate measure of hypercalcaemia and was the modality used primarily in the present analysis. The study cohort included patients with pHPT with a range of calcium levels, but the majority had mild hypercalcaemia. Only 3 of 19 patients aged 70 years or more had a calcium level that alone was considered an indication for parathyroidectomy according to the guidelines^20^. The MoCA score at baseline was related to age, but did not correlate with the level of hypercalcaemia. On the other hand, the baseline calcium level was identified as an independent predictor of long-term increase in MoCA score after parathyroidectomy in the whole cohort, as well as in the older age group in whom the long-term increase in MoCA score correlated with the decrease in calcium level.

The study has limitations. The cohort of elderly patients was relatively small. The presence of co-morbidities could have affected the results through changes in other conditions. Before inclusion, all patients had been selected for parathyroidectomy and the outcome could have been affected by patients’ expectations of improvement. The MoCA score also has limitations. Being primarily a screening assessment tool for detecting cognitive deficiencies, the score cannot constitute a basis for detailed neurodegenerative diagnostics. This study model permits limited conclusions about the causality of the cognitive processes related to pHPT.

The results of the present study, however, indicate that there are complex interrelationships between physical, psychological, and cognitive symptoms, and this test panel provides a useful way of predicting outcome after parathyroidectomy. The results emphasize the importance of paying greater attention to a wider assessment of domains that contribute to health-related QoL and the benefits that can be gained after parathyroidectomy, particularly in the elderly. Medical normalization of hypercalcaemia can, regardless of age, help in predicting outcomes after parathyroidectomy and is worthy of wider assessment as an aid to patient selection for surgery.

## Funding

Novo Nordisk Foundation

Amgen, Solna

Magnus Bergvall Foundation

Fredrik and Ingrid Thuring Foundation

Lisa and Johan Grönberg Foundation

## Supplementary Material

zraa029_Supplementary_Data
