# Supplementary material for: Prediction of cognitive response to surgery in elderly patients with primary hyperparathyroidism
Source: BJS Open. 2020 Dec 22;5(2):zraa029. doi: 10.1093/bjsopen/zraa029 (PMC7962724; doi:10.1093/bjsopen/zraa029)
Supplement: zraa029_Supplementary_Data [file zraa029_supplementary_data.docx]

**Table S1 Results of multiple regression analysis to identify independent predictors of long-term improvement in Montreal Congnitive Assessment score**
